# A Natural Mutation in Helix 5 of the Ligand Binding Domain of Glucocorticoid Receptor Enhances Receptor-Ligand Interaction

**DOI:** 10.1371/journal.pone.0164628

**Published:** 2016-10-13

**Authors:** Henry Reyer, Siriluck Ponsuksili, Ellen Kanitz, Ralf Pöhland, Klaus Wimmers, Eduard Murani

**Affiliations:** 1 Institute for Genome Biology, Leibniz Institute for Farm Animal Biology (FBN), Dummerstorf, Germany; 2 Institute of Behavioural Physiology, Leibniz Institute for Farm Animal Biology (FBN), Dummerstorf, Germany; 3 Institute of Reproductive Biology, Leibniz Institute for Farm Animal Biology (FBN), Dummerstorf, Germany; Universidad de la Laguna, SPAIN

## Abstract

The glucocorticoid receptor (GR) is a central player in the neuroendocrine stress response; it mediates feedback regulation of the hypothalamus-pituitary-adrenal (HPA) axis and physiological actions of glucocorticoids in the periphery. Despite intensive investigations of GR in the context of receptor-ligand interaction, only recently the first naturally occurring gain-of-function substitution, Ala610Val, of the ligand binding domain was identified in mammals. We showed that this mutation underlies a major quantitative trait locus for HPA axis activity in pigs, reducing cortisol production by about 40–50 percent. To unravel the molecular mechanisms behind this gain of function, receptor-ligand interactions were evaluated *in silico*, *in vitro* and *in vivo*. In accordance with previously observed phenotypic effects, the mutant Val610 GR showed significantly increased activation in response to glucocorticoid and non-glucocorticoid steroids, and, as revealed by GR-binding studies *in vitro* and in pituitary glands, enhanced ligand binding. Concordantly, the protein structure prediction depicted reduced binding distances between the receptor and ligand, and altered interactions in the ligand binding pocket. Consequently, the Ala610Val substitution opens up new structural information for the design of potent GR ligands and to examine effects of the enhanced GR responsiveness to glucocorticoids on the entire organism.

## Introduction

High concentrations of circulating glucocorticoids (GC) activate glucocorticoid receptor (GR) signaling in a variety of tissues to mediate processes like stress response, immune defense, and energy metabolism. These processes are thought to be regulated by GR to some extent independently from each other [1, 2] via different mechanisms of transcriptional activation and repression [3, 4]. Due to its potent anti-inflammatory action, GR is a target of many immunosuppressive drugs. However, the therapeutic effects of these drugs are often accompanied by undesirable side effects due to the pleiotropic function of the receptor and the cross-reactivity of steroid-based drugs. It is therefore imperative to reduce off-target effects by improving the selectivity of GC agonists [5, 6].

The ligand binding domain (LBD) of GR has been the focus of studies seeking to elucidate receptor-ligand interactions as well as GR transactivation, cytosol-to-nucleus transport, and interaction with chaperones [7]. The LBD of GR contains eleven α-helices and two β-sheets that form a three-layer protein structure [7]. Crucial for ligand binding are amino acid residues of helices (H) 3, 4, 5, 6, 7 and 10 that build specific interactions with distinct carbon atoms of the steroid. Specifically, as revealed for the murine GR, Gln570 (H3) and Arg611 (H5) are important for the interaction with the A-ring of the ligand [7]. Moreover, Asn564 (H3) establishes a key hydrogen bond with the C-ring of the ligand and, therefore, partially accounting for the specificity of nuclear receptors. Based on crystallographic analyses of the GR/dexamethasone structure, the residues Gln642 (H7) and Thr739 (H10) are described as important for the interaction with the 17α-hydroxy group and the 17β D-ring of the ligand, respectively. The sequence of the LBD is highly conserved between different vertebrate species. The distinct sequence differences to other members of the oxosteroid receptors mainly account for evolutionary adaptation of the GR for GC and consequently differentiation of its function, for instance from the osmoregulative effects of mineralocorticoid receptors (MR) [8]. The evolutionary changes in the architecture of GR-LBD including formation of specific side chain binding pockets for different ligands occurred at the expense of the stability of unbound receptors [9, 10]. This marginal stability limits the tolerance for mutations in GR and is the main reason that most mutations result in loss-of-function [6, 7, 11]. This raises the need to identify function-shifting mutations of GR-LBD preserving receptor activity which would provide better insights into LBD structure, specific receptor-ligand interactions, and global effects of selective GC agonists [6, 9, 12].

We recently discovered the first naturally occurring gain-of-function mutation in the GR-LBD: an Ala610Val substitution in helix 5 (H5) induced by the single nucleotide polymorphism (SNP) c.1829C>T (rs335303636) in porcine GR. This substitution increases the responsiveness of the porcine GR to dexamethasone, a synthetic GC, *in vitro*. On the genetic level this polymorphism is responsible for a major quantitative trait locus (QTL) for hypothalamus-pituitary-adrenal (HPA) axis activity, with high contribution to the genetic variance of cortisol and adrenal weights in pigs [13]. The aim of the present study was to uncover the molecular mechanisms responsible for the increased responsiveness of the mutated receptor. To this end, the effect of the Ala610Val substitution on GR activation and ligand selectivity, translocation, and interaction with GC was examined. The results presented here suggest that the mutation changes the structure of the ligand binding pocket, altering receptor-ligand interaction such that GR activity is increased, and provides insight into the helical interactions necessary for ligand recognition and binding.

## Materials and Methods

### Ethics statement

All samples analyzed in this study were collected *post-mortem*, from pigs raised and slaughtered in the context of pig production. These animals described herein are therefore not to be considered as experimental animals per se, as defined in EU directive 2010/63 and subsequent national application texts. Animal care and tissue collection was performed in compliance with the German Law of Animal Protection. The experimental protocol was approved by the Animal Care Committee of the Leibniz Institute for Farm Animal Biology, Dummerstorf, Germany.

### Transactivation assay

Porcine GR expression plasmids (pCMV-GRA610 or pCMV-GRV610) harboring the SNP *NR3C1* c.1829C>T (causing the Ala610Val substitution) were used for a transactivation assay in Cos-7 cells as previously described [13]. In brief, monkey kidney Cos-7 cells were seeded in 96-well plates at a density of 2.5×104 cells/well in DMEM (Invitrogen, Karlsruhe, Germany) with 10% FCS. Twenty-four hours after seeding, cells were co-transfected with 200 ng of expression vector, 100 ng of pGL4.36 reporter (Promega, Mannheim, Germany) and 2 ng of pRL-SV40 plasmid (Promega) according to the Lipofectamine 2000 protocol (Invitrogen). The reporter plasmid pGL4.36 expresses firefly luciferase under control of the glucocorticoid-inducible MMTV (Murine Mammary Tumor Virus Long Terminal repeat) promoter while the pRL-SV40 vector constitutively expresses the Renilla luciferase and was used to normalize for transfection efficiency. Cortisol, aldosterone, progesterone, and testosterone (all Sigma-Aldrich, Taufkirchen, Germany) were dissolved in pure ethanol and used to treat cells at concentrations ranging from 0.01 nM to 10 μM. Therefore, cell media was replaced by DMEM supplemented with respective steroid concentrations and 10% charcoal-stripped FCS (PAA Laboratories, Coelbe, Germany). Twenty-four hours after stimulation, firefly and Renilla luciferase activities were measured using the Dual-Glo Luciferase Assay System (Promega) in a DTX 880 Multimode Detector (Beckman Coulter, Krefeld, Germany).

Comparable overexpression of both GR variants in Cos-7 cells and the proportion of endogenous GR transcript expressed in Cos-7 cells were initially checked by real-time PCR using GR-specific primers GRf5 (CACCTGGATGACCAAATGACC) and GRr4 (AGGGTAAAGCCATTCTCTGCTC) and the Light-Cycler 480 SYBR Green I Master Kits on a Light-Cycler 480 Real-Time PCR System according to manufactor's instructions (Roche, Mannheim, Germany). Transcript abundance was corrected for the copy number of expression plasmid. Analyses revealed similar transcript abundance of both GR variants (5.17E+06 copies for Ala610 GR and 5.48E+06 copies for Val610 GR) and more than 13000 fold higher abundance of exogenous overexpressed GR compared to endogenous GR in Cos-7 cells under the same conditions.

For all steroids except testosterone, two separate experiments were performed in triplicate. Data were adjusted for transfection efficiency and the resulting luciferase ratios were normalized to cells that were treated with ethanol only. Fold-changes of transcriptional activity were compared using a generalized mixed linear model including fixed effects of genotype, hormone concentration, and the interaction of the two. Data were analyzed using SAS V9.3 software (SAS Institute, Cary, NC, USA). Least-square means were compared at each concentration and p-values were adjusted by Dunnett’s post-hoc test. Fold-change values were further adjusted to maximum relative luciferase induction and dose-response curves, EC50, and significance of EC50 differences were estimated using dose-response equation of GraphPad Prism 5 (GraphPad Software Inc, San Diego, CA).

### *In vitro* glucocorticoid receptor binding assay

A whole-cell GR binding assay was adapted from the protocol previously described for peripheral blood mononuclear cells (PBMC) [14]. Cos-7 cells were seeded in 10-cm dishes and T75 flasks at a density of 1.75x10^5^ cells/mL. After 24 hours in culture, cells were transfected with pCMV-GRA610 or pCMV-GRV610 using either 10 μg plasmid and 50 μL Fugene HD (Promega) for dishes or 15 μg plasmid and 75 μL Fugene HD for flasks. After another 24 hours in culture, media were replaced with serum-free DMEM to avoid contamination with steroids. One day later cells were pelleted, washed with PBS, and resuspended in DMEM supplemented with 10 mM HEPES buffer. Cell suspensions were incubated with [^3^H]-dexamethasone (specific activity 43 Ci/mM; Amersham Pharmacia Biotech, Freiburg, Germany) at six different concentrations (ranging from 1.5 to 50 nM) on a shaker at 37°C for one hour. Duplicate incubations were performed in the absence and in the presence of 500-fold molar excess of unlabeled dexamethasone (Sigma-Aldrich) to determine total and non-specific binding. After incubation, cells were washed with ice-cold PBS and centrifuged (10 min, 400 × g, 4°C) three times. Cell pellets were then resuspended in 100 μL PBS and mixed with 3 mL scintillation fluid (Rotiszint eco plus, Carl Roth GmbH, Karlsruhe, Germany). Radioactivity was measured in a spectral liquid scintillation counter (Tri-Carb 2900TR, Perkin Elmer Inc., USA) at 50% efficiency. The experiment was performed three times.

Saturation binding analysis included in nonlinear regression models implemented in GraphPad Prism 5 was used to fit binding curves to estimate the dissociation constant (K_D,_ mean ± SE), maximum specific binding (B_max,_ mean ± SE), and differences in these values dependent upon GR genotype.

### *In vivo* ligand binding assay

Animal care and tissue collection was performed in compliance with the German Law of Animal Protection. The experimental protocol was approved by the Animal Care Committee of the Leibniz Institute for Farm Animal Biology, Dummerstorf, Germany. Pigs used for sample collection were raised under standardized conditions in the performance test station Jürgenstorf (Germany) according to national regulations for performance testing (ZDS).

Pituitary glands were quickly removed from pigs after electronarcotization and exsanguination, snap-frozen, and stored at -80°C. Pigs were genotyped for SNP *NR3C1* c.1829C>T, and plasma cortisol concentrations were measured as previously described [13]. Samples from barrows homozygous for the wild-type allele or heterozygous were pooled, taking into account cortisol levels and relatedness of animals. Nine pools per genotype, each containing two to three samples, were analyzed. In total 40 pituitaries were used. Pituitaries from homozygous carriers of the alternative variant were not available due to its low frequency.

GR binding analysis was performed on the pooled samples as previously described [15, 16]. Briefly, tissues were homogenized in ice-cold 10 mM Tris-HCl buffer, pH 7.5, containing 12.5 mM EDTA, 10 mM sodium molybdate, 0.25 mM sucrose, and 5 mM dithiothreitol using an electric homogenizer (Ultra-Turrax, IKA, Staufen, Germany). The homogenate was centrifuged at 120,000 × g for 60 min at 0–4°C to isolate cytosol (supernatant fraction). GR binding was evaluated in saturation experiments using [^3^H]-dexamethasone over a concentration range of 0.2 to 24 nM. Non-specific binding was determined with a parallel incubation containing 500-fold excess of RU 28362 (kindly donated by Roussel Uclaf, France), which binds selectively to GR. The separation of bound from free ligand was performed by precipitation with dextran-coated charcoal and the receptor-[^3^H]-steroid complexes were counted in the Tri-Carb 2900TR scintillation counter at 50% efficiency. Protein concentrations for each pool were determined with bovine serum albumin as a standard [17]. Binding values were corrected for the amount of protein input. Individual binding curves for each pool were fitted using GraphPad Prism 5 as described above. Resulting K_D_ and B_max_ values were compared between genotypes using a mixed linear model implemented in SAS. The model included the effect of genotype and experimental batch.

### Cellular translocation of GR after stimulation with dexamethasone

Green fluorescent protein (GFP)-tagged expression vectors were generated to track subcellular distribution and movement of both GR variants. In brief, the EGFP region was amplified from EGFP-N1 (Clontech, Heidelberg, Germany), using primers EGFP_N_fw and EGFP_N_rev (Table 1) and the proofreading PrimeSTAR HS DNA Polymerase (MoBiTec, Göttingen, Germany). The product was inserted into the pCMV-Tag1 vector (Agilent Technologies, Waldbronn, Germany) using *Not*I restriction sites. The resulting plasmid was further digested with *Bam*HI and *Bgl*II restriction enzymes to insert *Bcl*I digested GR-alpha fragments. Therefore, GR-alpha fragments were amplified using cDNA of two individuals homozygous for the c.1829C>T SNP with primers GR_N-Flag_fw2 and GR_N-Flag_rev (Table 1). Orientation and integrity of both expression plasmids, pCMV-GFP-GRA610 and pCMV-GFP-GRV610, were verified by sequencing.

**Table 1:** Primer information.

Primer	Sequence 5´–3´
EGFP_N_fw	ATAATAGCGGCCGCCCACCATGGTGAGCAAGGGCGAGG
EGFP_N_rev	ATAATAGCGGCCGCCCTTGTACAGCTCGTCCATGCC
GR_N-Flag_fw2	ATAATATGATCAGACCCCAAGGAATCGCTGAC
GR_N-Flag_rev	ATAATATGATCATCACTTTTGATGAAACAGAAGTTTTTTG

Cos-7 cells were seeded in culture dishes (Greiner Bio-One, Frickenhausen, Germany) or 8-well chamber slides (VWR International, Hannover, Germany) for live cell imaging at a density of 1.6x10^5^ cells/mL in DMEM with 10% FCS and transfected one day later with pCMV-GFP-GRA610 or pCMV-GFP-GRV610 using a Lipofectamine:DNA-ratio of 3:1. Twenty-four hours after transfection, media were replaced with DMEM supplemented with 10% charcoal-stripped fetal calf serum (FCS) (PAA Laboratories). Before visualization by microscopy, media were replaced with phenol red-free DMEM supplemented with 5% charcoal stripped FCS, and the dish or slide to be analyzed was placed in a microscope incubator at 37°C and 5% CO_2_. Cells were stimulated with 100 nM dexamethasone (Sigma), and cellular localization of the GFP-GR fusion protein was examined using a confocal laser scanning head LSM 5 Pascal attached to an inverted microscope (Axiovert 200M, Carl Zeiss, Jena, Germany). Time series analyzer plugin (V2.0) of the ImageJ 1.47f software (National Institutes of Health, Bethesda, Maryland, USA) was used to analyze quantitative data from three independent transfection experiments with at least six observed chambers per genotype. Fluorescence intensity of the whole microscope image was used to correct for bleaching. To compare both genotypes, the average time to obtain half-maximal fluorescence intensities in a region of interest in the nucleus was estimated for at least four cells per dish (mean ± SE). In total 59 and 39 single cells were analyzed for Ala610 GR and Val610 GR, respectively.

### Modeling of the GR ligand-binding domain

Crystallographic data of the murine GR-LBD generated by Seitz *et al*. [12] were used to generate in silico models of the porcine GR-LBD employing WinCoot software (version 0.7.1) [18]. The porcine Ala610 and the Val610 GR structures are based on Research Collaboratory for Structural Bioinformatics Protein Databank (http://www.rcsb.org/pdb/) entries 3MNE and 3MNO, respectively. Both crystallographic datasets, 3MNE and 3MNO, were generated by Seitz *et al*. [12] using the stabilizing F608S mutation but differ for the Ala611Val substitution of murine GR corresponding to Ala610Val of porcine GR. Accordingly, the region of interest for assessing the effect of the Ala610Val substitution are represented by these structures. The porcine protein sequence (NCBI Accession # NP_001008481) was aligned to the murine ortholog to evaluate sequence differences between the LBD (amino acids 525 to 783 of murine GR) of both species. The region showed 97.3% sequence similarity to the porcine GR-LBD sequence. Protein sequences were adjusted by *in-silico* exchanging 21 amino acids, out of the 258 amino acid included in the LBD models, according to the porcine sequence (murine amino acid positions: 525, 529, 530, 557, 561, 567, 607, 621, 623, 624, 633, 640, 649, 653, 654, 656, 683, 739, 749, 766, 771). Protein geometry was validated by Ramachandran plot, and geometry, rotation, and density fit of amino acid residues were adjusted using the corresponding tools of the WinCoot software. Dexamethasone was fitted as ligand for both models and CCP4mg software [19] was used for rendering, graphic data output, and geometry measurements.

## Results

### Ala610Val substitution enhances responsiveness of GR to endogenous GC

**Fig 1:**
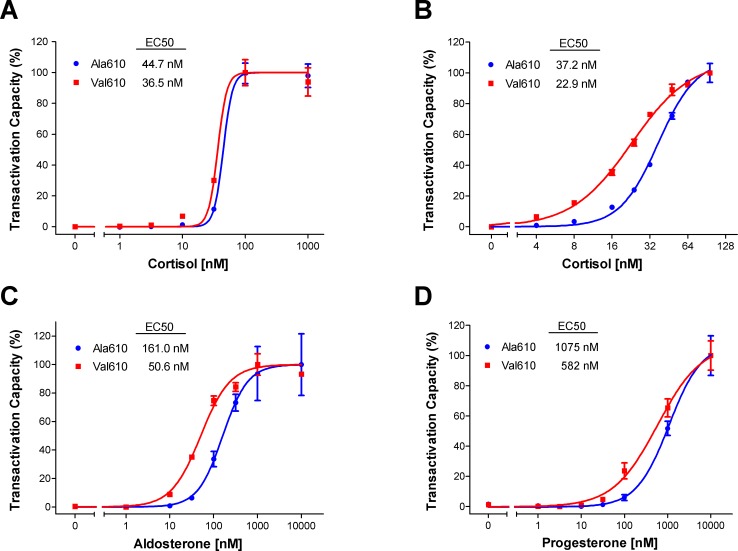
Transcriptional activity of alternative GR variants harboring the Ala610Val substitution after treatment with different concentrations of steroid. Presented are dose-response curves and EC50 estimations for the endogenous ligand cortisol in a wide (A) and narrow range (B). Additionally, the transactivation activity of aldosterone (C) and progesterone (D) is illustrated. The wild-type variant is labeled as Ala610 (blue) and the mutant GR variant as Val610 (red). Plotted are normalized means ± SEM of two separate experiments performed in triplicate.

Our previous studies of the Ala610Val substitution revealed marked differences between both receptor variants in transactivation activity after stimulation with the synthetic GC dexamethasone [13]. Because the effects of LBD mutations on the transactivation activity of GR may be ligand-dependent, we further examined transactivation activity of the alternative receptor variants after stimulation with cortisol, the endogenous GC. The wild-type Ala610 and the mutant Val610 receptor variants showed similar dose-dependent induction of a MMTV-driven reporter in the absence of cortisol and at maximal cortisol concentrations (S1 Fig). In contrast, the Val610 variant produced significant differences in transcriptional activation when stimulated with 1 to 30 nM cortisol (0.003<p<0.04) and exhibited 2.4-fold and 2.5-fold higher transactivation activity at cortisol concentrations of 10 nM and 30 nM, respectively. The substitution caused a significant shift in the estimated EC50 values (p = 0.0005), from 44.7 nM for the wild-type GR to 36.5 nM for the mutant GR (Fig 1A).

To increase the resolution of the dose-response experiment and to cover physiological cortisol concentrations, we examined cortisol-induced transactivation capacity in a narrower concentration range. In agreement with the results of the initial experiment, the dose-response curves differed significantly between Ala610 and Val610 receptors from 4 to 32 nM cortisol (0.0001<p<0.02) (S1 Fig). The Ala610Val substitution significantly increased responsiveness of GR to cortisol, as revealed by a 1.6-fold reduced EC50 (from 37.2 nM for wild-type GR to 22.9 nM for mutant GR; p<0.0001) (Fig 1B).

### Ala610Val substitution enhances responsiveness of GR to aldosterone and progesterone

All members of the oxosteroid receptor family have evolved to preferentially bind a certain ligand while also retaining some responsiveness to structurally-related steroids. Thus, we used transactivation assay to examine whether the Ala610Val exchange alters the sensitivity and selectivity of ligand binding for non-GC steroids. For aldosterone, the specific ligand of MR, the dose-response curves differed at concentrations ranging from 10 to 100 nM (0.006<p<0.02) (S1 Fig). Likewise, the progesterone transactivation assay revealed higher activity of the Val610 variant at concentrations of 30 nM (p = 0.02) and 100 nM (p = 0.03) (S1 Fig). Further, the kinetics of the progesterone curve indicated that the transcriptional response had not plateaued at the highest tested concentration of 10 mM. The estimated EC50 values were 3-fold (161 nM vs. 50.6 nM; p<0.0001) and 1.8-fold (1075 nM vs. 582 nM; p = 0.0021) lower related to the Val610 variant for aldosterone and progesterone, respectively (Fig 1C and 1D). Both receptor variants were unable to activate the MMTV-driven luciferase reporter after stimulation with testosterone, the endogenous ligand of androgen receptor (AR) (data not shown). Taken together, the results of the transactivation assay revealed that the mutant Val610 receptor variant exhibits an overall increased sensitivity but unchanged selectivity for the tested steroids.

### Ala610Val substitution shows no effect on GR translocation

**Fig 2:**
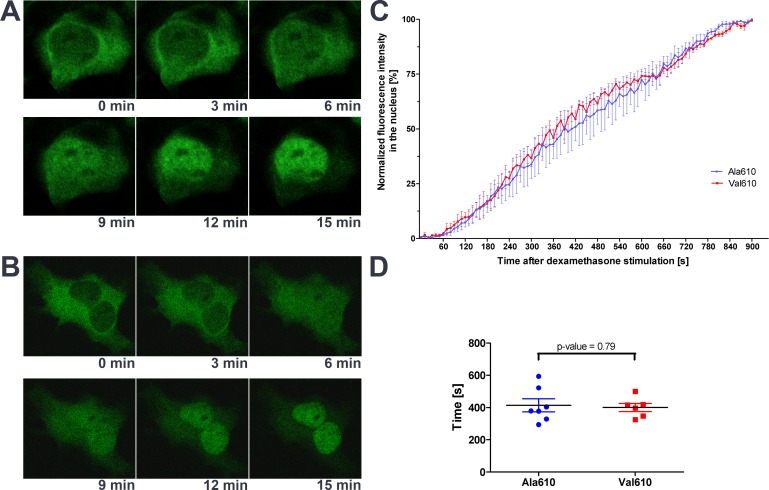
Cytosol-to-nucleus shuttle and subcellular localization of GFP-tagged GR variants. Cos-7 cells were transiently transfected with either the wild-type Ala610 (A) or the mutant Val610 (B) GR variant and stimulated with 10 nM dexamethasone to induce translocation. Representative images of time-series showing the same cells are presented (A and B). The increase in fluorescence intensity in the nucleus after dexamethasone stimulation (C) and the comparison of the estimated half-maximal translocation time between both GR variants (D) based on observations of three independent transfection experiments are depicted.

To elucidate the molecular mechanisms responsible for the increased responsiveness of the Val610 GR variant, we explored the influence of the Ala610Val substitution on structural and functional properties of the LBD. Depending on their position in the LBD, mutations are able to influence different functions. To test whether the Ala610Val substitution alters translocation of the activated receptor, GFP-labeled GR-variants were used to track the subcellular localization of GR after GC stimulation. In untreated cells, fluorescent fusion receptors were mainly localized in cytoplasm (Fig 2A and 2B). After stimulation with dexamethasone, both receptor variants fully translocated into the nucleus within 15 minutes. No differences were seen in the translocation rate of the wild-type Ala610 and the mutated Val610 GR variant (Fig 2C). Estimated half-maximal translocation time differed insignificantly (p = 0.788) with 414.3 ± 40.4 sec for Ala610 GR and 400.7 ± 25.1 sec for Val610 GR (Fig 2D).

### Ala610Val substitution affects the structure of the GR ligand binding domain

**Fig 3:**
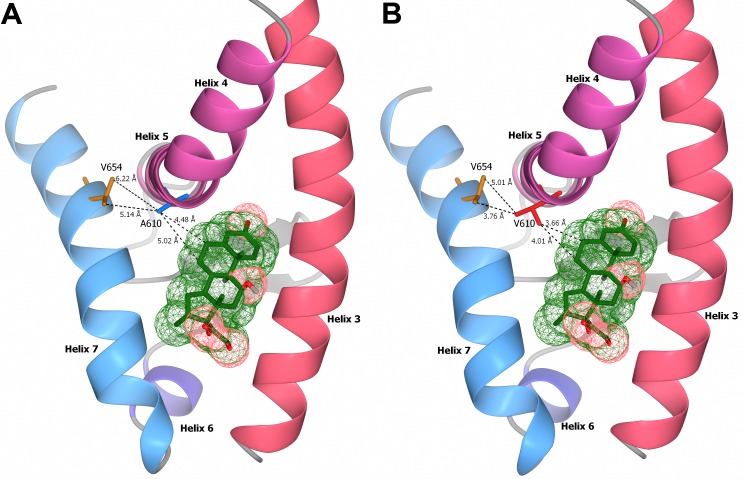
Stereogram of the interaction between glucocorticoid receptor ligand binding domain and dexamethasone. Structural models of helix 3 to helix 7 of the ligand binding domain of the wild-type Ala610 (A) and the mutated Val610 (B) receptor variants are shown. The distances of the alternative alanine (blue) and valine (red) residue at amino acid position 610 with the two methyl carbon atoms of Val654, and the carbon C-6 and C-7 positions of the ligand dexamethasone (green), are indicated in angstroms (Å).

Structural models of the interaction of the ligand binding pocket and dexamethasone were generated to investigate the influence of the Ala610Val amino acid exchange on the ligand binding ability of the GR. Comparison of LBD structures of both receptor variants revealed reduced binding distances between the valine residue of the mutant GR and the C-6 and C-7 position in the B-ring of dexamethasone (3.66 Å to C-6 and 4.01 Å to C-7; Fig 3B) compared to the wild-type receptor harboring the alanine residue (4.48 Å to C-6 and 5.02 Å to C-7; Fig 3A). Moreover, structural models indicated reduced distances in the H5-H7 interaction due to the Ala610Val substitution. The distances between the side chains of the alternative residue in H5 and the two methyl-carbon atoms of Val654 in H7 are 6.22 Å and 5.14 Å for Ala610, and 5.01 Å and 3.76 Å for Val610, respectively. This suggests intensified van der Waals forces between the affected amino acid residues in H5, H7, and the side chains of the ligand.

### Ala610Val substitution doubles ligand binding ability of GR

**Fig 4:**
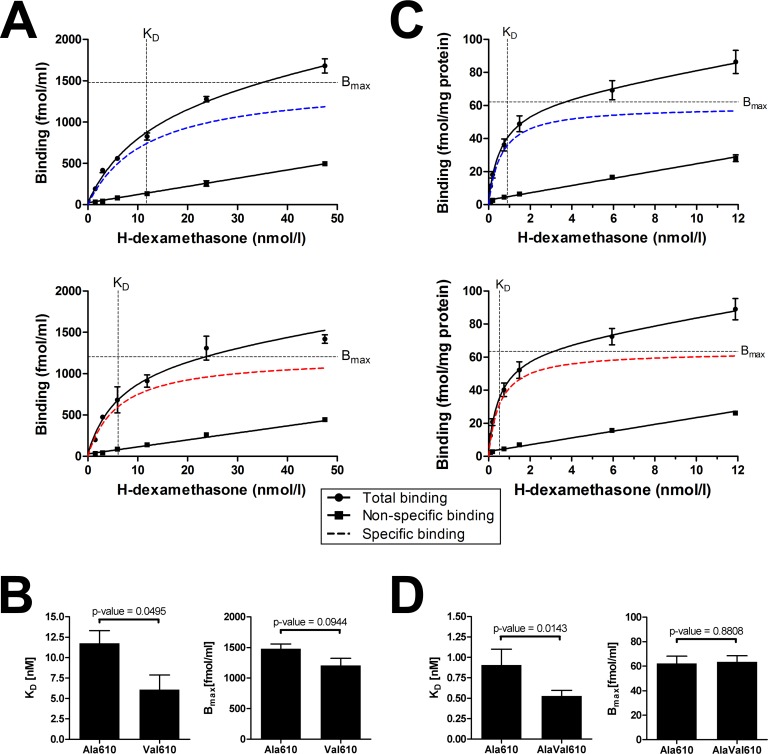
*In vitro* and *in vivo* glucocorticoid receptor binding assays. Dexamethasone binding assays were performed for transfected Cos-7 cells (*in vitro*; A and B) and for cytosolic fractions of pituitary glands (*in vivo*; C and D). For Cos-7 cells, specific binding curves (A) were plotted based on three independent experiments for Ala610 GR (blue) and Val610 GR (red). For pituitary glands, homozygous carriers of wild-type GR (Ala610 GR; blue) and heterozygous carriers of mutant GR (AlaVal610 GR; red) were analyzed (C). Dashed lines indicate the estimated dissociation constant (K_D_) and the maximum specific binding (B_max_), respectively. Comparisons of K_D_ and B_max_ between groups are illustrated for the *in vitro* (B) and in *in vivo* (D) binding assay as means ± SEM.

To experimentally verify the altered receptor-ligand interaction, whole-cell GR binding assays were performed *in vitro* in Cos-7 cells using allelic expression constructs. Both receptor variants showed specific binding curves depending on the ligand concentration and reached similar B_max_ values (Fig 4A; B_max_ = 1479 ± 79.7 fmol/ml for Ala610 GR and B_max_ = 1204 ± 118.1 fmol/ml for Val610 GR; p>0.05). Nevertheless, the mutant Val610 variant had a steeper slope of the binding curve compared to the wild-type receptor. Estimated K_D_ differed significantly (p<0.05): 11.76 nM for the Ala610 GR variant compared to 6.08 nM for the Val610 GR (Fig 4B). Consequently, the mutated receptor variant displayed a nearly two-fold higher binding affinity for GC *in vitro*.

To corroborate this finding, we performed additional ligand binding experiments *in vivo* using cytosolic extracts from the pituitary gland, an important target organ of GC (Fig 4C). In agreement with the *in vitro* studies, heterozygous carriers of the mutant GR showed a 1.7-fold increased affinity for GC compared to wild-type controls (Fig 4D; K_D_ = 0.906 ± 0.195 nM for Ala610 GR and K_D_ = 0.529 ± 0.068 nM for AlaVal610 GR; p<0.05). In contrast, B_max_ values were not significantly affected (62.2 ± 5.9 fmol/mg protein vs. 63.4 ± 5.0 fmol/mg protein; p>0.05), indicating that GR density in the pituitary is not affected by the Ala610Val substitution.

## Discussion

In this study we explored molecular mechanisms responsible for the previously documented gain-of-function phenotype of the Ala610Val substitution in the GR-LBD. This study demonstrated enhanced sensitivity of the mutated receptor variant to GC and other related steroids known to activate GR. The discovery of increased responsiveness of the mutated GR variant to non-GC steroids raises the question of whether physiologically relevant doses of those steroids are able to influence GR function *in vivo*. The estimated EC50 of the mutated GR variant for aldosterone (50.6 nM) is two orders of magnitude higher compared to circulating aldosterone concentrations in pig blood (about 150 pg/mL, equal to 0.04 nM [20]). Thus, the Ala610Val substitution is not in conflict with the separation of MR- and GR-specific signaling. Sequence analyses of PR and AR revealed a valine residue at the position corresponding to GR Ala610, whereas the common ancestral oxosteroid receptor is predicted to harbor an alanine at this position [21]. Thus, the Ala610Val substitution in GR resembles the evolution of the LBD of PR and AR at this specific position in H5. Nevertheless, we obtained no evidence for altered selectivity of the mutated GR variant; in particular, the mutated receptor variant remained insensitive to testosterone [22, 23]. Glucocorticoid receptors are able to bind progesterone [23], but showed no transactivation response to physiologically relevant progesterone concentrations [22]. Basal concentrations of progesterone are relatively low; however, the levels rise up to 60-fold during a normal menstrual cycle and more than 100-fold during pregnancy in humans [24, 25]. In consequence, the ubiquitously expressed GR harboring the Val610 variant may be able to mediate the effects of energy supply and immune suppression in response to circulating concentrations of progesterone. In fact, GCs were suggested to play a general role in implantation, proliferation, apoptosis, remodeling, and decidualization of endometrial cells [26].

As suggested by the lack of differences in subcellular distribution of GR and the cytosol-to-nucleus shuttle following activation by GC, the Ala610Val substitution seems to have no effect on the interaction of GR with importin channels [27]. Rather, the increased responsiveness of the mutated receptor appears to be a result of a reduced binding distance between the receptor and ligand, suggested by *in silico* models of receptor-ligand interaction and by the results of the ligand binding assay. The ascertained molecular and functional alterations induced by the substitution are in perfect accordance with its marked effects on the activity of the HPA axis. More specifically, the 1.5- to 2- fold enhancement in affinity and responsiveness of the valine receptor variant to GC accounts well for the observed 40–50 percent reduction in cortisol production [13]. Since so far only limited number of genetic variants contributing to the variation of quantitative traits (i.e. quantitative trait nucleotides, QTN) were identified and functionally characterized [28], these findings give important insights into the genetic architecture and biology of quantitative traits.

*In silico* structural models of the two alternative receptor variants illustrate the localization of the affected residues (Ala610Val) in H5 and the orientation of their side chain towards H7. The alternative hydrophobic amino acid establishes van der Waals contacts with carbon C-6 and C-7 positions of the B-ring of the steroid. The derived alterations of these contacts based on the Ala610Val substitution are in accordance with previous findings [12]. Interestingly, this receptor-ligand connection is also influenced by selective GC agonists. An additional single α-methyl group on the C-6 atom at the ligand level is known to increase its potency as shown by the comparison of synthetic GC methylprednisolone and prednisolone [29].

However, van der Waals forces are relatively weak protein forming forces [30] and it is worth considering whether solely the alteration of the binding distances between receptor and ligand is sufficient to show such considerable effects. More likely is that, in addition to altering the receptor-ligand interaction, the Ala610Val substitution also affects the architecture of the entire GR-LBD by altering H5-H7 connections. The structural analysis of Seitz *et al*. revealed that Ala611 (corresponds to Ala610 in porcine GR) interacts with Ile655 (corresponds to Val654 in porcine GR) in H7 and thereby influences the conformation of a structural loop between H5 and H6 formed by amino acids 621–628 of murine GR [12]. The rotation of Ile655 determines whether the structural loop 621–628 exists in an open, more variable, or a closed conformation [12]. Notably, Seitz *et al*. identified the Ala611Val substitution as having the greatest enhancement of the stability and solubility of GR-LBD in a high-throughput screen [12]. Comparative GC drug studies further illustrate that ligand-dependent displacement of the loop between H5 and H6 also contributes to differences in the pharmacological potency of GC agonists [5]. Furthermore, it was suggested recently that Ala605 in human GR (corresponding to Ala610 in the porcine GR) is critical for the rotational freedom of Met646 in H7 (Met651 in porcine GR) and plays a role in ligand selectivity [31]. The alanine residue was assumed to allow free rotation of Met651 while valine on this position sterically restricts the movement of the methionine, thus pointing to sterical interactions between residues in H5 and H7. The Met651 residue was, in turn, assumed to be important for the accessibility of the binding pocket occupied by the C-17 side chain of the steroid required for specific ligand recognition. Consequently, these studies point to the importance of interactions based on Ala610 for pharmacological studies of selective GC agonists [7, 31].

The impact of the Ala610Val substitution on the arrangement of H7 side chains could also influence ligand recognition, specificity, and selectivity based on interactions of H7 residues with steroids. In fact, two positions essential to changing the preference from mineralocorticoids to GC are Pro642 (Ser106Pro exchange of ancestral GR1; Pro637 in human GR) and Gln647 (Leu111Gln exchange of ancestral GR1; Gln642 in human GR) both located in H7 [32, 33]. These substitutions were crucial for the evolution of the GR and influence overall receptor specificity as well as sensitivity to GC [5, 33].

The protein model of the GR-LBD further illustrates that the Ala610Val substitution is one of the amino acid residues on the contact surface of H3, H5, and H7. Interestingly, previous studies established a critical role for H3-H5 interaction induced by point mutations in H5 of the LBD of GR (hGRMet604Leu corresponding to Met609 in the porcine GR), MR (hMRSer810Leu), and progesterone receptor (PR; hPRMet759Leu) in the regulation of ligand sensitivity and specificity [34, 35]. In their corresponding steroid receptors, these artificial mutations affect evolutionarily conserved residues directly adjacent to the position corresponding to the Ala610Val substitution [35]. In contrast to Ala610, the Met609 residue builds interactions with different positions of the steroid backbone and is located towards H3 instead of H7. Nevertheless, the Ala610Val exchange and the hGRMet604Leu substitution revealed strikingly similar effects at the functional level–in particular, enhanced binding affinity for GC and increased transactivation activity *in vitro* and at the phenotypic level [13, 35, 36]. We propose an expansion of the H3-H5 mechanism that includes the interaction of H3, H5, and H7, whereby H5 acts as connector with the amino acids in the contact surface mediating tension and flexibility between H3 and H7, and consequently influencing the shape of the ligand binding pocket. Especially the flexibility of these helices is highly important for the process of ligand binding due to the fact that the ligand pulls H3 and, to a lower extent, H7 in the center of the binding pocket while coming in contact with the GR [37]. Moreover, mutational studies of H3 and H7 in steroid receptors emphasize both H5-associated helices as crucial for ligand binding and transactivation [7, 38, 39]. Consequently, the considerable effects on receptor function caused by the Ala610Val substitution can be explained by impacting multiple factors based on H5, e.g., effects on the conformation of H3 and its interaction with activation function helix (H12) [40], an altered LBD stability towards loop 621–628 mediated by H7 [12], and the architecture of the ligand binding pocket influenced by the H5-mediated perturbation of H3 and H7 [7].

The demonstrated changes in GR-LBD architecture and the associated increase in steroid sensitivity caused by the Ala610Val substitution provide new insight into the role of H5 as a central connector of the ligand binding pocket. The Val610 GR variant is the first GR variant with GC hypersensitivity found to be robust and stable in a natural background. Consequently, the Ala610Val substitution opens up new structural information for the design of potent GR ligands and to examine effects of the enhanced GR responsiveness to glucocorticoids on the entire organism.

## Supporting Information

S1 Fig Data curves of *in vitro* transactivation of GR. The wild-type Ala610 (blue) and the mutant Val610 (red) variant were transiently transfected in Cos-7 cells and stimulated with cortisol in a wide (A), and in a narrow range (B) and with non-glucocorticoid steroids aldosterone (C) and progesterone (D). Plotted are means of fold induction of relative luciferase expression ± SEM of two separate experiments performed in triplicate. Significant differences (p<0.05) between both GR variants are indicated by an asterisk.
(TIF)
