# Supplementary figures and images for: A Natural Mutation in Helix 5 of the Ligand Binding Domain of Glucocorticoid Receptor Enhances Receptor-Ligand Interaction
Source: PLoS One. 2016 Oct 13;11(10):e0164628. doi: 10.1371/journal.pone.0164628 (PMC5063400; doi:10.1371/journal.pone.0164628)

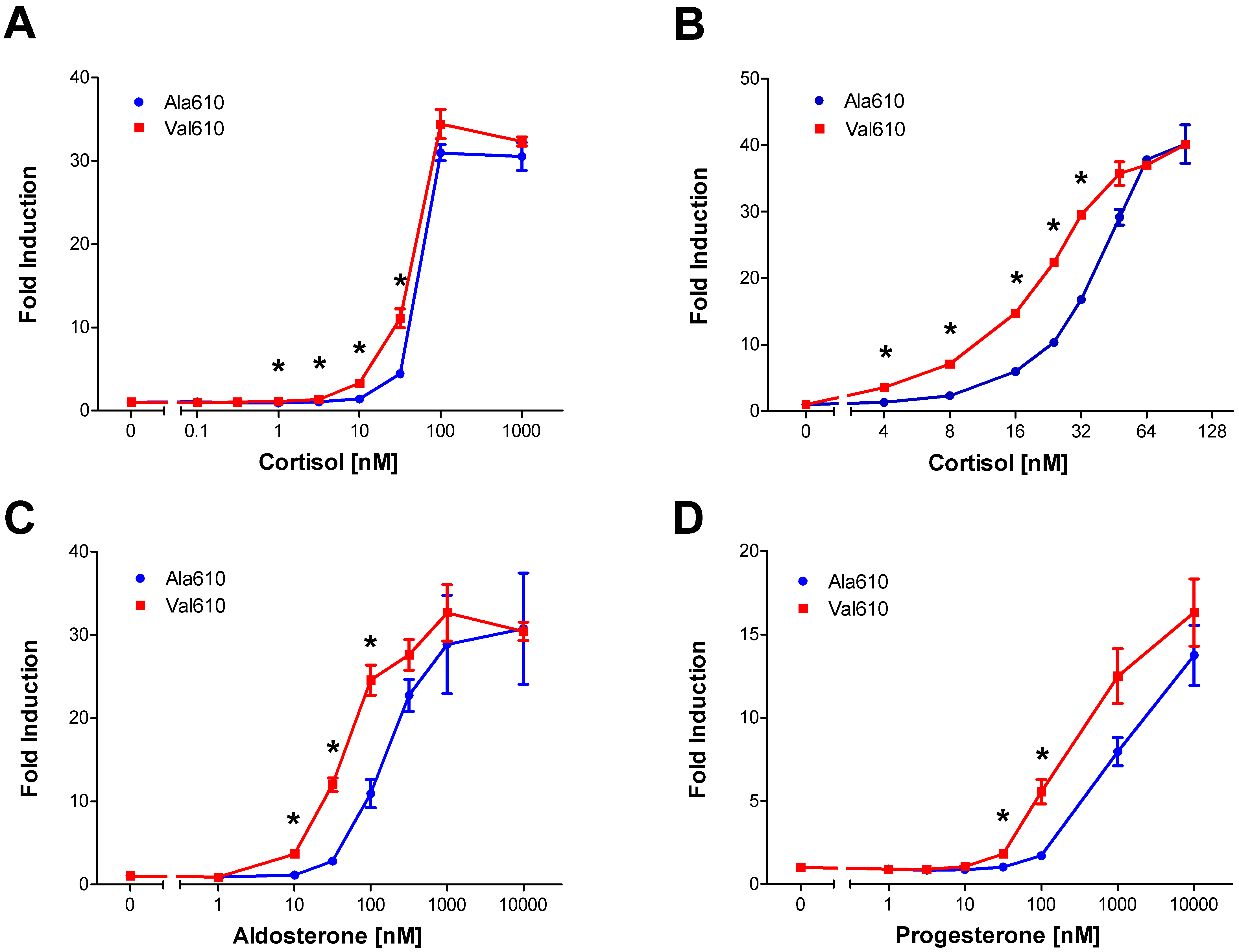

Supplement: S1 Fig — The wild-type Ala610 (blue) and the mutant Val610 (red) variant were transiently transfected in Cos-7 cells and stimulated with cortisol in a wide (A), and in a narrow range (B) and with non-glucocorticoid steroids aldosterone (C) and progesterone (D). Plotted are means of fold induction of relative luciferase expression ± SEM of two separate experiments performed in triplicate. Significant differences (p<0.05) between both GR variants are indicated by an asterisk. (TIF) [file pone.0164628.s001.tif]
